# On the Cow-Pox

**Published:** 1814-09

**Authors:** Richard Walker, S. Walker

**Affiliations:** Oxford; Oxford


*For the Medical and Physical Journal.*

*On the Cow-Pox*; by Mr. RICHARD WALKER, of Oxford.

IT will be apparent, that the contents of this paper were written at very different periods of time; the former part was written soon after the introduction of *vaccination*, and the latter, or concluding part, immediately before sending it to you.

The reader will perceive, in the course of this paper, an irrelevant kind of digression, which will be afterwards accounted for; and which, perhaps, may be not perfectly uninteresting. I have subjoined a summary view, in the form of a Table, of the ordinary progress of *inoculated small-pox*, which, with certain allowances, will serve likewise for *vaccination*, collected from a journal I kept of such cases.

The *cow-pox* I consider as a different modification, only, of the *small-pox*, and, though it may be, *ordinarily*, considered as a security against that disease, is yet less certain than the *small pox* itself. The circumstances of the *small-pox* being incident to every person, and, ordinarily, *once only*, and the greater probability of an attack of the *small-pox* after the *cow-pox*, than a second attack of the former, I would attempt to account for thus. I consider every person as having the essence or innate principle of the small-pox originally in the system, which remains *dormant*, unless matured and put into action by an exciting cause, of which, *infection* is the usual one; and to which every individual is so liable to be exposed, that no one has a reasonable expectation of escaping it.

Whenever the essence or innate principle of the disease is by any means put into action, it assimilates to its own nature a certain portion of the fluids, circulating in the system, more or less according to the grossness or purity of the habit, and throws out or expels them. If the whole of the original essence be expelled, or the system entirely purified of the disposition, which is ordinarily the case, the person will never be subject to a second attack.

The more complete the ferment or commotion in the system may have been at the time of the disease, the greater will be the probability of the whole having been thrown off, and *vice versa.*

Hence, although the cow-pox, properly passed through, may be a security *ordinarily* against the small-pox, yet, considering that persons pass through this disease, for the most part, without any eruption, and scarcely any discernible ferment or commotion in the system, I should be less surprised at an attack of the small-pox after the cow-pox, than a *second attack* of the small-pox itself.*

Since writing the preceding observations on *vaccination*, several instances have occurred, from unquestionable authority, (although a similar circumstance has not fallen immediately under my own notice,) of *natural small-pox*, in persons who had properly passed through *cow-pox.*

Although I am inclined to admit that these instances are too few to justify setting aside, or to invalidate the practice generally; yet it encourages me to mention a kind of prediction I made respecting the ultimate event of vaccination, at its introduction, so soon as I had ascertained the fact, as it then appeared to me, that no persons properly vaccinated need be apprehensive afterwards of an attack of small-pox. My opinion was, that, in consequence of the great satisfaction respecting security from inoculated *small-pox*, and in the present improved state of managing it, the little inconvenience or danger arising from it, compared with the insecurity after *vaccination*, arising from various causes, viz. the difficulty of procuring *fresh matter* at the *proper time*, and the probable failures and *mistaken security*, from its being imperfectly

* It has frequently occurred to me, what might be the effect of inoculating the cow with *small-pox matter;* whether such an experiment *has* been made, I do not know. This might have led to some further elucidation of the subject.

performed; performed; my sentiments respecting it were, that at a certain period it would be found to be a greater evil than benefit to the community, and that ultimately it would be entirely abandoned.

At the same time I wish it to be understood, that, provided these difficulties were removed, and that *vaccination* could be universally adopted and practised; notwithstanding the credit which is due to well-authenticated cases of *natural small-pox* occurring after *vaccination*, yet such instances will ever be so exceedingly rare as by no means to preclude pursuing this practice in preference to *small-pox inoculation.*

The inoculated small-pox is occasionally so mild as to pass off in as light a manner as is usual in vaccination, viz. with scarcely any apparent indisposition, and without any eruption. I have a case of this kind, at the time of writing this, in which I transferred matter taken from a person very full of natural small-pox. The inoculated part came progressively on, and was a mature pustule, on the *eleventh day.* It is worthy of notice, that another child, in the same family, passed through inoculated small-pox in a similar manner.

It is well known, that the smallest portion possible of the matter* of the *small-pox*, or *cow-pox*, in an active state, communicated to the absorbent or inhaling vessels, is sufficient to produce either of those diseases; but it is prudent in either instance, especially when a minute portion of the matter is trusted to, that the *puncture alone*, with the infected lancet, be not trusted to, but that the lancet be likewise wiped clean, as it were, of the matter, over or into the puncture.† I should not have descended to this observation, had I not known of occasional failures, (in young practitioners chiefly,) in the communication of these diseases, particularly the *cow-pox*, requiring a repetition of the operation, attributable, as I apprehend, to a neglect or omission of the above precaution. I never, myself, in any one instance, ever failed in communicating either *small-pox* or *cow-pox* by the above method, with the application of *fresh matter;* but I cannot say the same of *dried or preserved matter*, however carefully moistened, at the time of application.

That a recurrence of the *natural small-pox* in the same individual has happened, is, I presume, unquestionable; but

---

* By the term *matter*, I mean the *essence* of the disease, whether it be in a *serous* or *purulent state.*

† My constant method of performing this operation is by grasping the under part of the arm so as to pull the skin above upon the stretch, by which the puncture is more readily made, and the matter more perfectly inserted.

an an instance of this kind is an exceedingly rare phenomenon. The circumstance of *natural small-pox* happening subsequently to *inoculated small-pox*, is less rare; and the occurrence of the *natural small-pox* after *vaccine inoculation*, it must be admitted, is still less rare. These facts seem to accord with, or to confirm, the principle I at first laid down. By the following statement, however, of the proportion of such cases that may happen in each instance, it will be apparent, that upon this ground even *vaccination* may be considered as so far removed from any apprehension of subsequent *small-pox* as not to enter into the mind of any person.

I think I may venture to consider the following statement as nearly correct, or at least perfectly within the limits, viz. a recurrence of the natural small-pox once in ten million cases; natural small-pox occurring after inoculated small-pox once in one million cases; and of natural small-pox after vaccine inoculation once in ten thousand cases.*

The above statement may be considered as correct, when vaccine inoculation has been duly performed, and the subsequent effects, viz. the *proper progress of the pustule in the inoculated part;* and the *constitutional effect on the system*, which, though so slight as not to be apparent, oftentimes to ordinary persons, nevertheless, constantly or generally precedes or accompanies the highest state of inflammation of the inoculated part in some degree, and is a certain criterion of the disease having been properly passed through. It must be observed, however, that the absence of this latter circumstance is no proof of the negative; but this is a point to be determined on by the practitioner alone.

Moreover, it has ordinarily or generally happened, that an attack of natural small-pox, succeeding or subsequent to vaccination properly passed through, has been of a mild nature, diminished probably by the previous effect on the habit of the latter disease. Had I an only child, or any person whose life was most dear to me, I should vaccinate it myself, provided such matter were to be obtained fresh. If, however, there were danger of such person taking the natural small-pox, and no fresh vaccine matter to be obtained, I should not hesitate to inoculate with small-pox matter, well knowing that by attentive management I could almost to a certainty avert or obviate danger, or even vio-

* Some persons might be inclined to think this statement respecting the *cow-pox* too favourable; the certain fact is difficult to be obtained; but I think I am justified by reason and observation in stating, that, under my own care, did such an opportunity present itself, I should make the disproportion considerably greater.

lence, in the ensuing disease. I shall briefly observe here, that, in addition to the means ordinarily known and directed for this purpose, I lay much stress on keeping the patient, especially about the time of sickening, and the commencement of the eruption till its completion, as near as may be, to one uniform cool temperature, taking especial care to avoid all sudden transitions of heat and cold. I have frequently been able to attribute a load of pustules to a cause, I believe, but little thought of or attended to, viz. the circumstance of the patient, after having been exposed to cold air, particularly in the winter season, coming suddenly into a warm or rather a hot room, by which means the system becomes preternaturally heated, and excited by re-action.

So long, therefore, as vaccine matter is to be obtained in a proper state for inoculation, and the practice continued to proper persons, the mode by vaccination is unquestionably to be preferred; but from the difficulty of obtaining at present, even now, whilst the ardour for propagating this disease, inseparable from novelty, excites exertions, but which are far short occasionally of being adequate to the extraordinary call for it; I am apprehensive that my prediction before-mentioned may be in danger, at some future period, of being verified.*

It will be apparent, that the prediction above-mentioned was made soon after the introduction of vaccine inoculation. The prevention of its being fulfilled, as I heartily hope it may, will depend upon the continued zeal of the public in general, and the constant exertions of the faculty, viz. the general acquiescence of parents, and the zeal of the faculty in keeping up a constant supply of good matter; together with attention in communicating the disease, and watching the progress of it. For some time since small-pox in Oxford and its vicinity has become a rare occurrence.

I have lately met with a veteran in the profession, still retaining his proper judgment in other professional matters, who is still such a tenacious adherent of the old school, as to declare it to be his opinion, that every person is as incident to small-pox after vaccination as before, viz. that vaccination is of no avail whatever in preventing small-pox! Upon questioning him upon what data this opinion was founded, he declared he never had inoculated with vaccine matter,

---

* The confined nature of cow-pox, comparatively with that of inoculated small-pox, for furnishing matter, this being to be obtained from the inoculated part only, renders it necessary occasionally to send to London for it; from this cause failures likewise will sometimes arise, in consequence of the matter not being fresh.

and and that he never would; and, in fact, that he had never seen the cow-pox!

This is carrying prejudice against any novel mode of practice evidently too far. It is true my own experience has very much diminished my faith of its efficacy, from the same cause, I presume, which, as it appears, had previously entirely worn out the faith of the above-mentioned gentleman.

I am not surprised that any experienced practitioner should, at the first introduction of so extraordinary a novelty in practice as vaccination, being at the same time unacquainted with the established fact, that persons taking this disease from the cow, were rendered incapable of taking small-pox, should at first withhold his assent; but that such an unqualified dissent, in contradiction to the present general opinion, should at this time exist in the mind of any practioner, excited my astonishment.

*Oxford, Aug.* 4, 1814. S. WALKER.

*(The Editors have been obliged to postpone the remainder of this Article, which will be continued in our next.)*

